# A review of the scientific literature on experimental toxicity studies of COVID-19 vaccines, with special attention to publications in toxicology journals

**DOI:** 10.1007/s00204-024-03854-8

**Published:** 2024-09-03

**Authors:** Jose L. Domingo

**Affiliations:** Laboratory of Toxicology and Environmental Health, School of Medicine, Universitat Rovira i Virgili, Sant Llorens 21, 43201 Reus, Catalonia Spain

**Keywords:** COVID-19, Vaccines, Nonclinical studies, Experimental animals, Toxicity, Adverse effects

## Abstract

Since the reports of the first cases of COVID-19, in less than 5 years, a huge number of documents regarding that disease and the coronavirus (SARS-CoV-2), responsible for the infection, have been published. The tremendous number of scientific documents covers many topics on different issues directly related to COVID-19/SARS-CoV-2. The number of articles—including reviews—reporting adverse/side effects of the approved COVID-19 vaccines is considerable. A wide range of adverse/side effects have been reported in humans after COVID-19 vaccination: thrombotic events/thrombocytopenia, myocarditis/pericarditis, cutaneous reactions, immune-mediated effects, psychiatric adverse events, systemic lupus erythematosus, reproductive toxicity, and other miscellaneous adverse effects. In contrast, information on nonclinical studies conducted to assess the potential toxicity/adverse effects of the COVID-19 vaccines in laboratory animals, is comparatively very scarce. The present review was aimed at revising the scientific literature regarding the studies in laboratory animals on the toxic/adverse effects of COVID-19 vaccines. In addition, the investigations reported in those specific toxicology journals with the highest impact factors have been examined one by one. The results of the present review indicate that most nonclinical/experimental studies on the adverse/toxic effects of the COVID-19 vaccines and/or potential candidates showed—in general terms—a good safety profile. Only in some animal studies were certain adverse effects found. However, a rather surprising result has been the limited number of available (in the databases PubMed and Scopus) nonclinical studies performed by the companies that have been the largest manufacturers of mRNA vaccines in the world. It is assumed that these studies have been conducted. However, they have not been published in scientific journals, which does not allow the judgment of the international scientific community, including toxicologists.

## Introduction

Without a doubt, at the public health level, the world impact of the coronavirus disease (COVID-19), the infectious disease caused by the SARS-CoV-2 virus, has been huge and massive. From a scientific point of view, the COVID-19 pandemic has generated an incomparable—and unprecedented—number of documents/articles/papers published—both per year and globally—in a period of less than 5 years. On June 12, 2024, the PubMed (https://pubmed.ncbi.nlm.nih.gov/) database showed 432,135 results when COVID-19 was used as the search term, while 227,768 citations/abstracts were found using SARS-CoV-2. In turn, on the same date, SCOPUS (https://www.scopus.com/) collected 343,925 and 153,183 documents for COVID-19 and SARS-CoV-2, respectively. To put these figures in context, an interesting comparative example could be the use of “cancer” as a keyword in a search. The number of documents included in PubMed was (on the same date) 5,118,002, but this figure covers more than 200 years of cancer research in contrast to the approximately 4.5 years elapsed since the origin of the COVID-19 pandemic. The currently available documents on COVID-19 involve a long series of topics related to SARS-CoV-2/COVID-19. These topics include detection, diagnosis tests and transmission, symptoms, treatments, and sequelae of the disease, as well as severity and mortality, psychological consequences, co-morbidities, and other important issues, such as relationships of the infection with exposure to environmental pollution, nutrition, lifestyle, etc.

Among the numerous investigations conducted on SARS-CoV-2/COVID-19, an important research field concerns the vaccines assessed/used to prevent and/or alleviate the health effects of COVID-19. On June 13, 2024, PubMed showed 73,354 and 46,483 documents when “COVID-19 and vaccines” or “SARS-CoV-2 and vaccines” were respectively used as search terms. However, the available information in scientific journals on nonclinical studies carried out to assess the potential toxic/adverse effects derived from the administration of the vaccines approved by international organizations is limited. In PubMed, using “COVID-19 vaccine side effects” and “toxicity of COVID-19 vaccines”, the number of current documents was on that date 9684 and 491, respectively. Interestingly, most of these publications correspond to human data, with only a reduced number reporting results of nonclinical/animal studies. Due to the severity of the COVID-19 pandemic, regulatory bodies authorized important reductions in terms and stages to approve the use of new vaccines. Nevertheless, it is well established that preclinical safety evaluation is one of the key phases in the development of new products. That evaluation should predict the safety in humans of the clinical administration of the vaccines by detecting their potential toxicity in animals. According to the General Principles for the Technical Review of Preclinical Safety Evaluation of Biological Products for Prevention (Xu and Li 2024), the evaluation of the potential toxic reactions caused by vaccines should mainly include the following issues: (a) assessment of the direct damage to the body caused by potentially toxic components, (b) evaluation of the immune-related toxicity caused by the induced immune system, and (c) evaluation of the potential toxicity that could be caused by certain contaminants and residual impurities.

The number of articles—including reviews—reporting adverse/side effects of the approved COVID-19 vaccines that have been mostly administered to the population is considerable. Just as an example, here are some reviews covering a wide range of adverse/side effects that have been reported in humans after COVID-19 vaccination: thrombotic events/thrombocytopenia (Rosenblum et al. 2021; Marietta et al. 2022; Uzun et al. 2022; d’Almeida et al. 2023; Dalmia et al. 2023; Yasmin et al. 2023), myocarditis/pericarditis (Pillay et al. 2022; Chary et al. 2023a), cutaneous reactions (Gubernot et al. 2023; Bin Rubaian et al. 2023), immune-mediated effects (ElSawi and Elborollosy 2022), psychiatric adverse events (Ryoo et al. 2024), systemic lupus erythematosus (Cahuapaza-Gutierrez 2024), reproductive toxicity (Ma et al. 2023; Mukherjee et al. 2023), and other miscellaneous adverse effects (Meo et al. 2021; McColl et al. 2023; Chary et al. 2023b; Bitounis et al. 2024; Rasheed et al. 2024; Kanuri and Sirrkay 2024; Seyhan 2024). In contrast, the information—available in the scientific databases—regarding nonclinical studies on the potential toxicity/adverse effects of the COVID-19 vaccines, conducted in laboratory animals, is comparatively scarce. Taking the above into account, the present review was aimed at revising the scientific literature regarding the studies on the toxic/adverse effects of COVID-19 vaccines that have been carried out in experimental species of animals.

## Search strategy

For preparing this review, PubMed (https://www.ncbi.nlm.nih.gov/pubmed/) and Scopus (https://www.scopus.com) were used as scientific databases. The search was carried out using the following terms: “side effects of COVID-19 vaccines in experimental animals”, “toxicity of COVID-19 vaccines”, “toxicity of COVID-19 vaccines in animals”, “side effects of COVID-19 vaccines in rodents”, and “experimental toxicity of COVID-19 vaccines”. This first part of the search covered all documents published until June 18, 2024, either in toxicology journals or in journals of any other specialty. On the other hand, it can be expected that an important number of toxicological studies related to adverse/toxic effects of any new substance (not only vaccines) may be published in specific toxicology journals. Thus, considering this, the present review has been focused on reviewing those toxicology journals with the highest impact factors and prestige among the international community of toxicologists. The documents on the topic here reviewed, which have been published in the most relevant toxicology journals according to Clarivate (https://mjl.clarivate.com/home), were carefully checked one by one. PubMed was again used as the database for searching the documents published in each journal. The results are next presented.

## Studies on the adverse/toxic effects of COVID-19 vaccines published in non-specific toxicological journals

The following papers report the results of studies in which no toxic/adverse effects were observed in laboratory animals after administration of COVID-19 vaccines. Hassan et al. (2022) investigated the potency and toxicity of three PastoCoAd candidate COVID-19 vaccines, which were developed against SARS-CoV-2 using adenoviral vectors (containing the full-length S protein or the receptor-binding domains of SARS-CoV-2 S and N proteins). The adenoviral vector toxicity was assessed by single and repeated dose toxicity assays in Wistar rats that received intramuscular injections (100 µL) of adenovectors. The potential toxicity was also examined in mice and guinea pigs, while local tolerance was tested in guinea pigs. No significant toxic effects were found in the brain, lung, heart, and liver, as well as in muscles—at the injection site—in both species of animals used in that study. In turn, Abdoli et al. (2022) determined the efficiency and safety of an inactivated whole-virus SARS-CoV-2 candidate vaccine (BIV1–CovIran vaccine) in various species of animals: BALB/c mice, Guinea pigs, Wistar rats, New Zealand White rabbits, and non‐human primates (Rhesus Macaque). For the toxicology evaluation, the following assays were carried out: abnormal toxicity tests, single dose toxicity and local tolerance studies, repeated dose toxicity study, and rabbit pyrogenicity test (in rabbits). Clinical examination, clinical pathology, macroscopic evaluation of injection site, as well as histopathology of brain, liver, kidney, spleen, heart, gonad, site of administration and inguinal lymph node, did not show adverse effects due to the intramuscular injection of the vaccine. On the other hand, Banihashemi et al. (2022) examined in four animal models (Syrian hamsters, mice, guinea pigs, and New Zealand white rabbits) the safety and efficacy of a combined intramuscular/intranasal COVID-19 vaccine candidate named RAZI-COV PARS. This is a recombinant-based vaccine where the subunits of S1, S2, and open-state S-Trimer proteins of SARS-CoV-2 are encoded into the mammalian expression vector PK001 to produce recombinant proteins. The safety evaluation included the potential toxic effects following repeated doses of the vaccine and the pyrogenicity assay. The results indicated that none of the vaccinated animals of the four species showed changes in general clinical observations/indicators, body weight and food consumption, hematological and serum analyses, and pathological examination of various organs. In another study carried out by Shanmugaraj et al. (2022), the protective efficacy, safety, and toxicity of Baiya SARS-CoV-2 Vax 1 (a plant-derived SARS-CoV-2 subunit vaccine) was evaluated in mice, rats, and cynomolgus monkeys. The results showed that intramuscular injections of the vaccine were well tolerated. It did not cause adverse clinical symptoms or toxic effects on internal body organs, as well as on physiological functions of the animals under the conditions of that study (even at the highest tested dose: 50 µg Baiya SARS-CoV-2 Vax 1). Leon et al. (2022) assessed in captive black-footed ferrets the safety, immunogenicity, and anti-viral efficacy of a subunit SARS-CoV-2 vaccine candidate. The CoV spike protein, particularly the receptor binding domain, was immunogenic and could successfully protect immunized animals against challenge with SARS-CoV-2. No morbidity or mortality were observed. Moreover, despite viral replication and shedding in the upper respiratory tract for up to 7-day post-challenge, no other signs of clinical disease were found in vaccinated or naive animals.

An absence of adverse effects of COVID-19 vaccines—or candidates—has been reported after vaccination of laboratory rhesus monkeys (Oh et al. 2023a) with the BNT162b2 vaccine (BioNTech/Pfizer), or in rhesus monkeys after intradermal injection of the SARS-CoV-2 inactivated vaccine (Vero Cells) (Yang et al. 2023). Also, the safety assessment of RQ3013 (a broad-spectrum mRNA vaccine against SARS-CoV-2 variants) conducted in mice, hamsters, and nonhuman primates did not show adverse/toxic effects (Tan et al. 2023). Good immunogenicity and safety were also observed following administration to pigs (Moros et al. 2023), mice (Barreiro et al. 2023), and cynomolgus macaques (Prenafeta et al. 2023) of PHH-1 V. PHH-1 V is a COVID-19 vaccine candidate based on a protein comprising the receptor binding domain fusion heterodimer, including the B.1.351 and B.1.1.7 SARS-CoV-2 variants. Recently, Köse et al. (2024) reported that single or repeated injections of the SARS-CoV-2 vaccine (Vero cells) into mice did not show adverse/toxic effects on the animals’ overall clinical health, performance abilities, and kidneys.

## Studies published in toxicology journals

### About Clarivate and journal impact factors (JIF)

In Clarivate’s Journal Citation Reports of 2022 (published in 2023), the section of Toxicology included 24 journals belonging to quartile 1 (JIF Q1: the top 25% of journals in that section in the list based on their impact factors, IF). The contents of those journals regarding the published documents on COVID-19 vaccines specifically, or about COVID-19 in general, have been examined one by one. Only those documents reporting studies on experimental/laboratory animals are discussed. Based on my long experience of more than four decades working in toxicology—as a researcher and professor—I would like to indicate that, independently of the metrics of the journal (mainly the IF), the most popular journals for toxicologists, where most of them publish the results of studies with animals (or also “in vitro” investigations), have been examined. Consequently, the current review has included not only the journals belonging to the Q1 list, but also other journals listed as Q2.

### Articles published in Clarivate Q1 journals

To date, using PubMed as a database, the following Q1 journals (Toxicology section) have not published any articles reporting studies conducted in experimental/laboratory animals that could be directly related to the assessment of the potential toxic effects of the vaccines administered for preventing SARS-CoV-2 infection. The number of documents (named “a”) in which COVID-19 is a keyword, as well as the number of documents (named “b”) related to experimental/laboratory studies of the potential toxicity of COVID-19 vaccines, is next summarized (a, b), going from the journal with the highest IF to the one with the lowest IF. These are the specific journals: Environmental Health Perspectives (36, 0), Particle and Fibre Toxicology (2, 0), Journal of Toxicology and Environmental Health-Part B-Critical Reviews (0, 0), Ecotoxicology and Environmental Safety (31, 0), Cell Biology and Toxicology (4, 0), Reviews of Environmental Contamination and Toxicology (0, 0), Critical Reviews in Toxicology (0, 0), Inflammopharmacology (122, 19), Mutation Research-Reviews in Mutation Research (3, 0), Chemico-Biological Interactions (43, 0), Nanotoxicology (2, 0), Toxics (46, 0), Aquatic Toxicology (3, 0), Journal of Exposure Science and Environmental Epidemiology (27, 0), Environmental Toxicology (4, 0), Environmental Toxicology and Pharmacology (10, 0), and Expert Opinion on Drug Metabolism and Toxicology (8, 0). The information on the potential adverse/toxic effects of COVID-19 vaccines published in the remaining Q1 journals is next summarized.

Annual Reviews of Pharmacology and Toxicology is the journal occupying the first position in Clarivate’s section of Toxicology. This prestigious journal in the fields of pharmacology and toxicology has published only two review papers on COVID-19, but they are not closely related to the experimental toxicity of vaccines for SARS-CoV-2 infection (Sivaraman et al. 2021; Pandamooz et al. 2022). The second journal in the Q1 list is Drugs. Although this journal is not included among the “classic” toxicology journals, in PubMed, Drugs shows 42 documents in which COVID-19 is a keyword. However, only 3 of these papers give some information—more or less—related to vaccines, although it is mainly focused on the treatment of the disease. In a review article, Lamb (2021) summarized the milestones in the development of BNT162b2 (Comirnaty®, BioNTech/Pfizer). This is a lipid nanoparticle-formulated, nucleoside-modified mRNA vaccine designed for the prevention of SARS-CoV-2 infection. The BNT162b2 mRNA COVID-19 vaccine received conditional approval in Switzerland in December 2020 for active immunization to prevent COVID-19 in people aged 16 and over. However, while in her review, Lamb (2021) reported data on studies about adverse events of that vaccine, information on experimental animals is absent. In a recent review, Zhu et al. (2024) have discussed the effectiveness, advantages, and disadvantages of authorized COVID-19 vaccines. Since 2020, approximately 400 vaccines have been developed, with more than 13.4 billion doses of COVID-19 vaccines administered worldwide. In relation to this, Zhu et al. (2024) have stated that—like for any new type of vaccine—safety and effectiveness are also the two most important indicators for evaluating potential COVID-19 vaccines. The authors have indicated that despite significant progress in the development and study of COVID-19 vaccines, various issues must be still resolved to develop a safer, more effective, longer-lasting, and broader-spectrum vaccine against COVID-19. In their article, information on the potential toxic effects of the vaccines conducted in laboratory animals is not included. In another recent review article, Syed (2024) has summarized the milestones in the development of ensitrelvir fumaric acid (Xocova^®^), which led to a standard approval in Japan (November 2002) for SARS-CoV-2 infection. Again, no information about experimental toxic effects is reported.

Archives of Toxicology is currently one of the most reputed journals for researchers in toxicology. It has published 24 documents on COVID-19, from which 7 are directly related to COVID-19 vaccines. The first document is a Letter to the Editor that was published by Arand (2021). At that time, he suggested that “it might be informative to reanalyze the clinical trials and/or the post-marketing data of the adenovirus-based vaccines to gain a better understanding of the actual correlation between infectious units-based dosing and protection efficacy.” The first experimental study on the topic reviewed here was published in Arch Toxicol by Madar-Balakirski et al. (2022). These investigators assessed the safety profile of the rVSV-ΔG-SARS-CoV-2-S vaccine. A series of nonclinical safety, immunogenicity, and efficacy studies were conducted in four animal species: mice, hamsters, rabbits, and pigs. In comparison to (unvaccinated) controls, there were no treatment-related mortalities, nor any noticeable systemic or local clinical signs. Differences in hematology and biochemistry parameters were unremarkable, while there were no adverse histopathological findings. It was concluded that the rVSV-ΔG-SARS-CoV-2-S vaccine was safe and immunogenic in the nonclinical studies conducted in the four species of animals. In a subsequent study conducted by the same research group (Rosner et al. 2022), the potential toxicity, local tolerance, immunogenicity, and biodistribution of the vaccine rVSV-ΔG-SARS-CoV-2-S were investigated in New Zealand white rabbits. Systemic clinical signs, local reactions, body weight, body temperature, food consumption, ophthalmology, urinalysis, clinical pathology, C-reactive protein, viremia, and antibody levels were monitored, while gross pathology of organs/tissues was also carried out for biodistribution and histopathological evaluation. The observed changes were multifocal minimal myofiber necrosis at the injection sites, as well as increased lymphocytic cellularity in the iliac and mesenteric lymph nodes, and in the spleen. These changes were related to the inflammatory reaction elicited and were correlated with a trend for recovery. Based on those results, it was concluded that the rVSV-ΔG-SARS-CoV-2-S vaccine was not associated with major local or systemic adverse effects. Therefore, it was considered safe. In turn, Dai et al. (2022) evaluated the immunogenicity, biodistribution, and in vivotoxicological profiles of AdC68-19S (a recombinant chimpanzee adenovirus serotype 68 (AdC68) vector-based vaccine encoding the full-length spike protein of SARS-CoV-2) in rat and rhesus macaque. Rats and rhesus macaques were intramuscularly injected with AdC68-19S up to 2 0^11^vp/dose or 4 0^11^vp/dose, two or three times, with a 14-day interval period, respectively. AdC68-19S was well tolerated in both species. Only a mild and reversible interstitial inflammation of muscle was observed at the injection site, but no signs of systemic and/or local toxicity were found. It was concluded that AdC68-19S might induce an effective immune response with a good safety profile. On the other hand, Oh et al. (2023b) evaluated in white rabbits the potential toxicity, local tolerance, and immunogenicity of the intradermal vaccine candidate pGO-1002 (a non-viral DNA vaccine that expresses both the spike and ORF3a antigens of SARS-CoV-2). Mortality and clinical signs, body weight and food consumption, skin irritation, ophthalmology, body temperature, urinalysis, clinical pathology, as well as gross observations and histopathological evaluation were monitored/performed. Changes in lymphocytes, as well as local inflammatory changes, were observed, which were considered to occur due to the vaccine or the intradermal injection. However, systemic toxic changes were not found following vaccine administration. It was concluded that the pGO-1002 vaccine was safe and effective under the experimental conditions of the study. In another preclinical evaluation of a potential vaccine for COVID-19, Park et al. (2023) assessed in rats, rabbits, and dogs the toxicity profile of HuVac-19. It is a subunit vaccine of SARS-CoV-2 that utilizes the receptor-binding domain as an antigen. Single- and repeated-dose studies were carried out. Although in the general toxicity and safety pharmacology evaluations, HuVac-19 was found not to be associated with major systemic adverse effects, some changes were observed. Thus, the repeated-dose toxicity studies in rats and rabbits showed transient alterations in hematological and serum biochemical parameters in the adjuvant and/or vaccine groups. However, those changes were reversed—or potentially reversible—after the recovery period. Temporary reversible changes in absolute and relative organ weights were also detected in the prostate of rats and in the thymus of rabbits. On the other hand, a gross exam of the injection sites in animals treated with the adjuvant and HuVac-19 showed discoloration and foci. In turn, histopathological examination showed granulomatous inflammation, inflammatory cell infiltration, and myofiber degeneration/necrosis. Nevertheless, the inflammatory response was local, not being associated with other toxicological effects. In a recent study conducted in the same laboratory, Park et al. (2024) investigated the genotoxicity and safety pharmacology of the rVSVInd(GML)-mspSGtc COVID-19 vaccine using micronucleus and comet assays. In addition, neurobehavioral, body temperature, respiratory, and cardiovascular assessments were conducted in rats and beagle dogs. Only marginal changes in the body temperature, respiratory rate, heart rate, and ECG parameters were noted in rats and dogs, but these changes recovered within 24 h. There were no significant changes in the neurobehavior of the animals, while there were no increases in the number of bone marrow micronucleated polychromatic erythrocytes or liver DNA damage.

According to the aims and scope of the journal Inflammopharmacology, that journal would not seem to fit too well in Clarivate’s section of Toxicology. However, as indicated on the website of the journal, one of the seven main interest areas is: “The Safety and efficacy of Non-prescription (OTC) and prescription NSAIDs and Analgesics”. Probably, that is the reason for its inclusion in the Toxicology section of Clarivate. Thus, the search in PubMed, using COVID-19 as a keyword, found 122 documents. Among these, 19 are—more or less—related to COVID-19 vaccines. However, most of these articles are out of the scope of the current review. Although including vaccines, they mainly refer to possible preventive options for COVID-19 (Sebok and Gyires 2023; Vitiello et al. 2021), or to the immunogenicity and safety in humans of some COVID-19 vaccines (Batibay et al. 2022). Only a review article includes some comments on the safety of mRNA vaccines (Vitiello and Ferrara 2021). These authors stated that—at the time of their review—clinical data were showing very good efficacy of mRNA vaccines, as well as acceptable safety against COVID-19. These authors also indicated that further clinical data were still urgently needed to clarify important questions on vaccination. In that review, there were no data regarding experimental/laboratory studies with the vaccines.

The journal Toxicology has published (PubMed) 8 papers in which COVID-19 is a keyword. Only one study was aimed at investigating the safety of a potential COVID-19 vaccine. In a repeated-dose toxicity and local tolerance study in rats, Oliva-Hernández et al. (2022) assessed the safety profile of FINLAY-FR-02. It was a vaccine candidate against SARS-CoV-2, whose active ingredient is the recombinant receptor binding domain monomer (mRBD) conjugated to tetanus toxoid. Animals were monitored for the following milestones: clinical signs, pain, and body weight; water and food consumption; temperature; muscular diameter at the injection site; dermal irritability; blood chemistry; immunological response and immunotoxicity; and histopathology. The results did not show clinically relevant changes, pain and local effects, adverse systemic toxicological changes, or an increased mortality. Therefore, Finlay-FR-02 was considered safe in rats.

According to PubMed, using COVID-19 as a keyword, the journal Food and Chemical Toxicology has published 36 documents on the topic. Among these, 5 are directly related to SARS-CoV-2 vaccination. In the first paper, Huang et al. (2021) reported the results of a study aimed at evaluating in rats the toxicity and potential adverse effects of an inactivated SARS-CoV-2 vaccine (Vero cells) after multiple intramuscular injections. The following parameters were monitored: water and food intake, body weight, hematology, and serum biochemistry, CD4^+^ T cell and CD8^+^ T cell determination, and antinuclear and neutralizing antibody detection. Gross and histopathological examinations of various organs were also carried out. The results did not show any significant signs of obvious toxic effects. In February 2022, Food Chem Toxicol published a call for papers on the potential toxic effects of COVID-19 vaccines (Domingo 2022). That call encouraged researchers to submit the results of their investigations on the potential adverse effects of COVID-19 vaccines in order to clarify what the toxicological risks—if any—of the already approved vaccines could be. It was stated that research on long-term toxic/adverse effects should undoubtedly be an issue of special interest. The results of that call were certainly very limited, with just a few submissions. Seneff et al. (2022) published an article on the innate immune suppression by SARS-CoV-2 mRNA vaccinations, in which they remarked that the immune response to the mRNA vaccines was very different from that to a SARS-CoV-2 infection. These authors questioned the safety of most administered mRNA vaccines, based mainly on various aspects. Firstly, the subversion of innate immunity, primarily via suppression of IFN-α and its associated signaling cascade. Second, the dysregulation of the system for both preventing and detecting genetically driven malignant transformation within cells, and the consequent potential for vaccination to promote those transformations. Third, mRNA vaccination potentially would disrupt intracellular communication carried out by exosomes. According to Seneff et al. (2022), these disturbances could be related to neurodegenerative diseases, myocarditis, immune thrombocytopenia, Bell’s palsy, liver disease, impaired adaptive immunity, as well as impaired DNA damage response and tumorigenesis. That was a controversial article, which—to date—has generated eight comments in PubMed (available at: https://pubpeer.com/publications/7275D7B99CDB301C67743F214AF751?utm_source=Chrome&utm_medium=BrowserExtension&utm_campaign=Chrome). The most critical response to the article of Seneff et al. (2022) was carried out in a Letter to the Editor (initially rejected by the Editor of Food Chem Toxicol due to various concerns, but surprisingly finally accepted by a new Editor of that journal) written by Barrière et al. (2023). The authors of that Letter raised a series of important concerns about the validity of the article by Seneff et al. (2022). A list of bibliography misunderstandings was presented in the Letter, which concluded by stating that there was no evidence of the increased risk of cancer after COVID-19 mRNA vaccination. On the other hand, Xu et al. (2022) investigated if active small molecules contained in Ganoderma lucidum (G. lucidum) could effectively inhibit COVID-19 through network pharmacology. G. lucidum is a functional food used in traditional Chinese medicine, which can be used to improve the low immune function in various diseases. After conducting several in vitro assays, it was found that lucidenic acid A, an active component of G. lucidum, could inhibit the binding of the hACE2 receptor with the spike protein to prevent SARS-CoV-2 infection. It was shown that the in vitro inhibitory effect of lucidenic acid A on hACE2 binding activity had a good inhibitory effect on hACE2 with IC50 2 μmol/mL.

The journal drug safety is also included among the Q1 journals in the list of the Clarivate section of Toxicology. However, in the aims and scope, it may be read that Drug Safety is the official journal of the International Society of Pharmacovigilance. It publishes documents on issues, some of which can be directly related to the toxicological effects of drugs. The number of documents on COVID-19 (PubMed) is 63, with 35 of them concerning COVID-19 vaccination. Nevertheless, most papers are related to adverse effects/events in humans (some recent examples: Raethke et al. 2023; Walton et al. 2023; Kaur et al. 2024; Gordillo-Marañón et al. 2024a, b; Caplanusi et al. 2024), while none of them has reported results on toxic/adverse effects studies conducted in experimental/laboratory animals. Toxins (Basel) is the last Q1 journal in the Toxicology list of Clarivate. This journal has published 21 documents in which COVID-19 is a keyword. Notwithstanding, only a review article by Gupta and Pellett (2023) is very slightly related to the main topic of the current review. In that article, the authors discussed the recent developments in vaccine designs, including, of course, all related to the mRNA vaccines used to protect against SARS-CoV-2 during the COVID-19 pandemic. While the authors commented on some of the adverse effects reported in humans, there is not any information regarding preclinical studies conducted in laboratory/experimental animals.

### Articles published in Q2 toxicology journals and other specific toxicology journals

In addition to the Q1 journals included in the toxicology list of Clarivate, the present review has also examined the documents published in some Q2 toxicology journals, as well as other toxicology journals that are well appreciated and respected by toxicologists. Most of these journals have a wide diffusion in the field. They regularly publish results of general in vivo/in vitro toxicological studies. In the quartile 2 (Q2, Clarivate), and following the decreasing impact factors (IFs) in the list, Chemical Research in Toxicology is the first Q2 toxicology journal. In PubMed, 12 documents were found when COVID-19 was used as a keyword, although only one article has a direct relationship with the safety of the COVID-19 vaccines (Barda and Cerny 2021). These authors discussed the safety of two approved mRNA vaccines (Pfizer/BioNTech and Moderna), concluding that the experience with both vaccines demonstrated high efficacy and safety in the first 2 months. Notwithstanding, no studies carried out in experimental animals were discussed in that paper. The next examined journal was Toxicology and Applied Pharmacology. Eighteen documents with COVID-19 as a keyword have been found in that journal. However, only two articles (Baralic et al. 2020; Kalarikkal and Sundaram 2021) have a minimal relationship with COVID-19 vaccination, although without including experimental data. Toxicological Sciences, another well-known and reputed journal, is the official journal of the US Society of Toxicology. However, it has published only eight documents in which COVID-19 is a keyword. Only two of them (Yanagida et al. 2021; Babenek et al. 2022) report some information on COVID-19 vaccines, but again, none of the articles is directly related to experimental/laboratory assays on the potential toxic effects of the COVID-19 vaccines. In turn, Toxicology Letters has published three papers on COVID-19, but none of these is related to vaccination.

From my personal point of view, one of the most informative articles on the topic reviewed in the current paper has been published in Regulatory Toxicology and Pharmacology. Although PubMed cites only 6 documents published in that journal with COVID-19 as a keyword, one of these, published by Schilder et al. (2023), summarizes relevant information. These authors examined the results of nonclinical toxicity studies of various SARS-CoV-2 vaccines, which were produced with different manufacturing technologies. The article was mainly focused on Repeated Dose Toxicity (RDT) and Developmental and Reproductive Toxicity (DART) studies. The European Medicines Agency (EMA) nonclinical assessment reports for market authorizations were also evaluated. It was stated that the information was not always publicly available, although excerpts were published in the European Public Assessment Reports, being in the public domain. Schilder et al. (2023) found that there were no evident adverse/toxic effects in the nonclinical SARS-CoV-2 vaccine toxicity studies. Most observed findings were immunostimulatory effects, which together with their reversible nature, could be considered non-adverse/toxic. The authors noted that due to the low frequency of observations—outside of expected pharmacological inflammatory responses—and their species-specificity, there would be limited added and translational value of product-specific nonclinical studies for SARS-CoV-2 vaccines. In conclusion, in vivo animal toxicity testing would show only a limited value in establishing the safety of vaccines, being the vaccine-induced effects strongly species-specific. Another interesting article published in the same journal was written by Baldrick (2022). This author analyzed whether the number and types of studies normally needed for regulatory agency authorization of vaccines, biologicals (mAbs), and small molecules for patient use had been reduced for COVID-19 therapies. Six vaccines, 7 monoclonal antibodies, and 4 small molecule therapies were included in the study. It was noted that for 6 compounds showing potential utility against SARS-CoV-2, general toxicity studies along with PK studies for mAbs and small molecules, and reproductive toxicity testing for vaccines and small molecules, as well as genotoxicity testing for small molecules, were carried out. The analyzed data suggested that generally, the number of nonclinical studies was appropriate. On the other hand, among the 3 documents published in the journal Neurotoxicology regarding COVID-19, one of them (da Luz et al. 2022) gives data that could have been of interest for the development of COVID-19 vaccines. Thus, da Luz et al. (2022) evaluated in two strains of mice the toxicity of the SARS-CoV-2-derived peptide (called PSPD-2002). It was found that exposure of mice to PSPD-2002, a peptide derived from the spike protein of SARS-CoV-2, induced alterations involving redox and cholinesterasic homeostasis in the brains of animals. It was especially evident in C57Bl/6 mice, whose IBRv2 value was higher than that found in Swiss mice. The results of that investigation showed the importance of evaluating not only the susceptibility of different mammal species to viral infection (as well as their roles in the dissemination of COVID-19) but also their responses to exposure to viral particles.

Reproductive Toxicology has published 17 documents on COVID-19, from which 4 are related to the safety of COVID-19 vaccines. Bowman et al. (2021) carried out a study in rats aimed at evaluating the non-clinical developmental and reproductive toxicity of BNT162b2 (Pfizer vaccine). A dose of 30 μg mRNA/dose (which was equivalent to > 300 times the human dose expressed on a mg/kg basis) was administered intramuscularly to 44 female rats, 21 and 14 days prior to mating, as well as on gestation days 9 and 20. One-half of the animals were subjected to cesarean section and full fetal examination at the end of gestation. The other half of the rats were allowed to deliver, being monitored to the end of the lactation period. No adverse effects of the vaccine on female mating performance, fertility, or any ovarian or uterine parameters were observed. There were also no toxic/adverse effects on embryo-fetal development, postnatal survival, growth, or the development of the offspring at the end of lactation. In turn, Stebbings et al. (2021) assessed the potential adverse effects of AZD1222 (ChAdOx1 nCov-19, popularly known as the AstraZeneca COVID-19 vaccine) on the fertility and reproduction of female mice during the embryofetal development phase, as well as on postnatal development during the littering phase. No vaccine-related adverse/toxic effects on female reproduction, fetal/pup survival, and fetal external, visceral, and skeletal alterations were observed. No significant differences with controls in physical development, and no abnormal gross pathology findings were found in pups and dams. On the other hand, Dubé et al. (2022) evaluated in rats the pre- and postnatal effects of AS03, a developed recombinant plant-derived virus-like particle vaccine candidate for COVID-19 (CoVLP). AS03-adjuvanted CoVLP was intramuscularly administered to female rats before cohabitation 8 and 22 days prior to mating, and on gestation days 6 and 19. The potential adverse effects of AS03-adjuvanted CoVLP were evaluated on pregnant animals and on embryofetal development, parturition, and lactation. The development of the F1 offspring up to weaning was also evaluated. The results did not show adverse effects of the vaccine candidate on the fertility and reproductive performance of the vaccinated rats. Moreover, no evidence of adverse effects on embryofetal development/teratogenicity or the pre-weaning development of the F1 offspring was observed. The last article published in Reproductive Toxicology regarding maternal–fetal outcomes of COVID-19 vaccines is a review written by Piekos et al. (2022), which was focused on providing recommendations for vaccination against COVID-19 during pregnancy. It was concluded that, based on the reports published until that time, approved vaccines were safe and effective in pregnant people.

Clinical toxicology is defined as an international journal aimed at publishing research on the various aspects of clinical toxicology, including the diagnosis and treatment of poisoning. This journal has published 45 articles on COVID-19, but in fact only one (Tadfor et al. 2023) has reported adverse reactions after administration of COVID-19 vaccines. However, that observational study was based on the reports in humans collected by the New Mexico poison center hotline, not including data in laboratory animals. The report included 638 adverse drug reaction cases, which implicated these brands: Pfizer BNT162b2 (46.6%), Moderna mRNA-1273 (43.41%), and Janssen Ad26.COV2.S (8.78%). It was concluded that most complaints concerned systemic reactions, with reaction differences among vaccine brands (between the first and second doses for some effects) and selected recurrent events. In turn, the Journal of Applied Toxicology has published only two papers on COVID-19, reporting one of them the results of a non-clinical safety assessment of an mRNA COVID-19 vaccine candidate. In that study, Broudic et al. (2024) evaluated in rabbits and mice the potential toxicity and biodistribution of MRT5500 (an mRNA vaccine encoding for the full-length of the SARS-CoV-2 spike protein), which is delivered by lipid nanoparticles containing a novel ionizable lipid, Lipid-1). In a toxicity study, rabbits received 3 intramuscular injections of MRT5500 at 3 week intervals, followed by a 4 week observation period. In an exploratory biodistribution study, mice received a single intramuscular injection of an mRNA encoding luciferase encapsulated in an LNP containing Lipid-1. The results of both studies showed that the MRT5500 vaccine was safe and well-tolerated, while the biodistribution data demonstrated that the components of the MRT5500 vaccine formulation were predominantly observed at the injection site and in the draining lymph nodes. On the other hand, among the 21 documents related to COVID-19 published in the journal Immunopharmacology and Immunotoxicology, only one of them concerns potential adverse effects of SARS-CoV-2 vaccines. Thus, Huang et al. (2022) evaluated in guinea pigs whether repeated intramuscular administration (three times, once every other day) of inactivated SARS-CoV-2 vaccine (Vero cells) could induce active systemic anaphylaxis, while the potential degree of severity was also evaluated. Twenty-four animals were divided into four groups. The inactivated SARS-CoV-2 vaccine group received inactivated SARS-CoV-2 vaccines (challenge dose: 200 U in 1 mL/animal). On days 14 and 21 after the final priming injection, a challenge test was conducted. One-half of the guinea pigs in each group received intravenous injections with twice the dose and volume of the substance used for immunization. No deaths, abnormal reactions at the injection site, clinical symptoms, or differences in body weight were found between the control groups and the group receiving the inactivated SARS-CoV-2 vaccine. Allergic reactions were not observed either.

The journal Toxicology In Vitro has published three documents regarding COVID-19. One of these (Viskupicova et al. 2023) reports the results of a screening of scientific databases for compounds with activity against SARS-CoV-2 M^pro^ (main protease). The candidates were subsequently evaluated for their potential anti-inflammatory and/or antibacterial properties. Although some low-toxic inhibitors with antibacterial and anti-inflammatory effects were identified, it was also noted that side effects of some drugs could worsen COVID-19 treatment, with specific toxicities being key properties for drug candidates. Ten documents on COVID-19 are cited in PubMed corresponding to the journal Toxicologic Pathology. Among these, two papers report data of studies carried out in laboratory animals. Ramot et al. (2021) evaluated the systemic toxicity and local tolerance of the TKSB01 plasmid in Sprague Dawley rats, delivered intramuscularly and followed by electroporation. The TKSB01 plasmid is a vaccine (pTK1A-TPA-SpikeA, named COVID-eVax) that encodes a secreted monomeric form of SARS-CoV-2 S protein receptor-binding domain. Rats were sacrificed 2 days and 4 weeks after the last injection (days 30 and 57, respectively). Neither mortality nor signs of toxicity were found, including injection site reactions. Histopathological examination showed muscle fiber necrosis associated with subchronic inflammation at the injection sites. Specifically, it occurred at day 30, but with a clear trend for recovery at day 57. In a subsequent study, Ramot et al. (2022) assessed the systemic toxicity and local tolerance of the C1-cell-expressed receptor-binding domain (C1-RBD) SARS-CoV-2 vaccine in New Zealand white rabbits, following repeated intramuscular injections of the compound. All animals were monitored for systemic clinical signs and local reactions, body weight and body temperature changes, blood for clinical pathology, urine analysis, and measurements of food consumption during the study period. Ten animals from each treatment group were sacrificed 24 days after the first injection, while the remaining 10 rabbits in each group were killed 42 days after the first injection. The results did not show local or systemic toxicity. It was concluded that the C1 SARS-CoV-2 RBD vaccine candidate, which was not associated with any relevant systemic adverse effects, would be safe following four repeated vaccination (intramuscular injections) sessions. Finally, two “classic” toxicology journals, Basic and Clinical Pharmacology and Toxicology and Human and Experimental Toxicology, have not published any articles directly focused on the potential toxicity of COVID-19 vaccines, with 17 and 3, respectively, being the number of documents on COVID-19 included in PubMed for these journals.

In addition to the documents found in the Clarivate Q1 and Q2 toxicology journals, other journals not specifically aimed at publishing toxicological—or closely related—studies, various articles reporting the results of nonclinical investigations that focused on assessing potential toxic/adverse effects of COVID-19 vaccines were found in PubMed/Scopus. The following articles respond to that search. Li et al. (2022) investigated in Balb/c mice whether there were differences in the cardiac pathology induced by intravenous (IV) or intramuscular (IM) injections of the BNT162b2 mRNA COVID-19 vaccine (Pfizer/BioNTech) in comparison to 0.9% saline injections. That study was carried out considering that post-vaccination myopericarditis had been reported to occur potentially after immunization with COVID-19 mRNA vaccines. After IV or IM injections of the vaccine or 0.9% saline (controls), clinical manifestations and histopathological changes were assessed. Moreover, tissue mRNA expression, as well as serum concentrations of cytokines/chemokines and troponin, were measured at different time points. It was found that IV, but not IM, administration of the COVID-19 mRNA vaccine induced a rapid onset of multifocal myopericarditis accompanied by elevated serum troponin, cardiomyocyte degeneration, as well as changes in necrosis and apoptosis. Adjacent inflammatory infiltrates of mononuclear cells, interstitial edema, and visceral pericardial calcification were also observed. In turn, Zirkenbach et al. (2023) evaluated the potential toxic/side effects and potential side effects of COVID-19 vaccination in different mouse strains. The mouse strain A/JOlaHsd, which is very susceptible to the induction of autoimmune diseases, and the mouse strain C57BL/6, which is known to be more resistant, received the mRNA vaccine BioNTech. No adverse effects related to inflammation or heart function were observed in mice after mRNA vaccination. The results were independent of age, gender, and the strains (more or less susceptible to the induction of experimental myocarditis). Notwithstanding, in the experiments with vaccination and immune checkpoint inhibitor treatment, some animals showed a low elevation of cardiac troponins in serum, as well as low scores of myocardial inflammation. Also related to potential cardiac side effects, recently Schreckenberg et al. (2024) investigated the effects of mRNA-based vaccines mRNA-1273 (Spikevax, Moderna) and BNT162b2 (Comirnaty, Pfizer/BioNTech) on the function, structure, and viability of isolated adult rat cardiomyocytes over a period of 72 h. The results showed that both vaccines induced functional disturbances in isolated cardiomyocytes, which corresponded pathophysiologically to cardiomyopathy. The observed ryanodine receptor (RyR2) impairment, as well as sustained protein kinase activation, could significantly increase the risk of acute cardiac events. It was concluded that although both mRNA vaccines were effective in preventing COVID-19 and its possible sequelae, the risk–benefit ratio of this kind of vaccine should be re-evaluated considering their hidden potential cardiotoxic effects.

A summary of the results of some of the most relevant studies discussed above is shown in Table 1.

**Table 1:** Studies on the adverse/toxic effects of COVID-19 vaccines (or candidates) published in toxicology journals (Clarivate Q1 and Q2)

Journal	COVID-19 vaccine or candidate*	Animal species	Main results about safety	References
Arch Toxicol	rVSV-ΔG-SARS-CoV-2-S	Mice, hamsters, rabbits, and pigs	No treatment-related mortalities, nor any noticeable systemic, or local clinical signs were observed	Madar-Balakirski et al. (2022)
Arch Toxicol	rVSV-ΔG-SARS-CoV-2-S	New Zealand white rabbits	The vaccine was not associated with major local or systemic adverse effects. It was considered safe	Rosner et al. (2022)
Arch Toxicol	AdC68-19S	Rats and rhesus macaques	No signs of systemic and/or local toxicity were found. AdC68-19S would induce an effective immune response with a good safety profile	Dai et al. (2022)
Arch Toxicol	pGO-1002	Rabbits	Changes in lymphocytes, and local inflammatory changes were observed, which were considered to occur due to the vaccine or intradermal injection. However, systemic toxic changes were not found following vaccine administration	Oh et al. (2023b)
Arch Toxicol	HuVac-19	Rats, rabbits, and dogs	Transient alterations in hematological and serum biochemical parameters in the adjuvant and/or vaccine groups were noted. Those changes were reversed -or potentially reversible- after the recovery period	Park et al. (2023)
Arch Toxicol	rVSVInd(GML)-mspSGtc	Rats, beagle dogs	Only marginal changes in the body temperature, respiratory rate, heart rate, and ECG parameters were observed. The changes recovered within 24 h	Park et al. (2024)
Toxicology	FINLAY-FR-02	Rats	The results did not show clinically relevant changes, pain, local effects, adverse systemic toxicological changes, or increased mortality	Oliva-Hernández et al. (2022)
Food Chem Toxicol	Inactivated SARS-CoV-2 vaccine (Vero cells)	Rats	The monitored parameters, including various analyses and gross and histopathological examinations did not show any significant sign of obvious toxic effects	Huang et al. (2021)
Regul Toxicol Pharmacol	Review article on nonclinical toxicity studies of various SARS-CoV-2 vaccines produced with different technologies	Several species	Most observed findings were immunostimulatory effects, which together with their reversible nature, could be considered as non-adverse/toxic. The vaccine-induced effects are strongly species-specific	Schilder et al. (2023)
Neurotoxicology	PSPD-2002	C57B1/6 J and Swiss mice	Alterations involving redox and cholinesterasic homeostasis were found in the brains, being especially evident in C57Bl/6 J mice	da Luz et al. (2022)
Reprod Toxicol	BNT162b2	Rats	No adverse effects of the vaccine were seen on female mating performance, fertility, or on ovarian and uterine parameters. There were no toxic/adverse effects on embryo-fetal, postnatal survival, growth, and on the development of the offspring at the end of the lactation	Bowman et al. (2021)
Reprod Toxicol	AZD1222	Mice	No adverse effects were observed on female reproduction, fetal/pup survival, and fetal external, visceral, and skeletal alterations. No differences with controls were noted on physical development, while no abnormal gross pathology findings were found in pups and dams	Stebbings et al. (2021)
Reprod Toxicol	AS03-CoVLP	Rats	No adverse effects of the vaccine candidate on the fertility and reproductive performance of the rats were noted. There was no evidence of adverse effects on embryofetal development/teratogenicity, or pre-weaning development of the F1 offspring	Dubé et al. (2022)
J Appl Toxicol	MRT5500	Rabbits and mice	MRT5500 was safe and well-tolerated. The biodistribution data demonstrated that the components of the vaccine formulation were predominantly observed at the injection site and in the draining lymph nodes	Broudic et al. (2024)
Immunopharmacol Immunotoxicol	Inactivated SARS-CoV-2 vaccine (Vero cells)	Guinea pigs	No deaths, abnormal reactions at the injection site, clinical symptoms, or differences in body weight were found in comparison to controls	Huang et al. (2022)
Toxicol Pathol	TKSB01 plasmid	Rats	Neither mortality, nor signs of toxicity were found, including injection site reactions. Histopathological exam showed muscle fiber necrosis associated with subchronic inflammation at the injection sites	Ramot et al. (2021)
Toxicol Pathol	C1-cell expressed receptor-binding domain (C1-RBD)	New Zealand white rabbits	The results on the adverse/toxic effects study did not show signs of local or systemic toxicity	Ramot et al. (2022)

*Details on the specific COVID-19 (or candidate) vaccines can be found in the text of this manuscript

## Conclusions and discussion

The current review was aimed at examining the results of nonclinical studies on the potential toxicity/adverse effects of COVID-19 vaccines and/or possible candidates that protect against SARS-CoV-2 infection. Only the research conducted in experimental/laboratory animals has been discussed here. In the scientific literature, there is a tremendous number of available documents concerning COVID-19/SARS-CoV-2. It includes specific documents on the clinical efficacy and safety of COVID-19 vaccines (and candidates). However, information regarding nonclinical/experimental studies on their potential toxicities is comparatively very scarce.

In various sectors of the world’s population, and from minute zero of the mass vaccination to prevent SARS-CoV-2 infection, there has been concern about the efficacy of the current vaccines, but very especially about their potential long-term side effects. This concern includes the adverse/toxic effects of the mRNA vaccines, an issue that has generated doubts not only in some segments of the general population, but even in a part of the scientific community. In an interesting review article on mRNA vaccines, Banoun (2023) questioned whether the mode of action of these vaccines should classify them as gene therapy products (GTPs), with everything that classification would entail. Based on the adverse events reported in pharmacovigilance databases, Banoun (2023) suggested studying the long-term expression, integration into the genome, transmission to the germline, passage into sperm, embryo/fetal and perinatal toxicity, genotoxicity, and tumorigenicity of the COVID-19 mRNA vaccines. The author highlighted that the long-term safety monitoring of GTPs is required over various years, while for vaccines, it is generally only performed over a few weeks. According to Banoun (2023), this should not be acceptable, given the persistence of the drug product and the expressed protein. It would explain why, regarding specifically the potential toxic/adverse effects derived from the administration of the mRNA vaccines (approved by international organizations), the available information in scientific journals is certainly very limited. In relation to this, a rather surprising result of the present review has been the very scarce number of available (in PubMed and Scopus) nonclinical studies performed by the companies that have been the largest manufacturers of mRNA vaccines in the world. If, as I assume, safety and toxicity studies of this kind of product were carried out, why the results were not published in scientific journals? It would have allowed the data to be subjected to the judgment of the international scientific community, including toxicologists. Probably, it would have helped to reduce some concerns in certain sectors. Finally, sticking specifically to the current scientific documents available (in PubMed and Scopus), the present review indicates that—in general terms—most nonclinical/experimental studies on the adverse/toxic effects of the COVID-19 vaccines and/or potential candidates showed a good safety profile. Only some studies conducted in animals reported certain adverse effects. These include changes in lymphocytes and local inflammatory changes in rabbits (Oh et al. 2023b), alterations involving redox and cholinesterasic homeostasis in brains of C57Bl/6J mice (da Luz et al. 2022), and transient alterations in hematological and serum biochemical parameters, as well as marginal changes in body temperature respiratory rate and heart rate of rats and dogs, which were reversed after the recovery period (Park et al. 2023, 2024).

## Data Availability

Data are available from the author on request.
